# A systematic scoping review of peer motivational climate in youth sports and physical activity: a bi-decennial update

**DOI:** 10.3389/fpsyg.2026.1635666

**Published:** 2026-03-11

**Authors:** Keonyoung Chung, Marcelo Cabral, Nikos Ntoumanis, Spyridoula Vazou

**Affiliations:** 1 Department of Kinesiology, Michigan State University, East Lansing, MI, United States; 2 Danish Centre for Motivation and Behaviour Science, University of Southern Denmark, Odense, Denmark; 3 School of Sport, Exercise, and Rehabilitation Sciences, University of Birmingham, Birmingham, United Kingdom

**Keywords:** adolescent, children, peer influence, psychosocial, social agents, social interactions

## Abstract

It has been 20 years since the term “peer motivational climate” was first conceptualized and measured. The purpose of this systematic scoping review was to (i) quantify the literature on the study characteristics and methodologies of peer motivational climate research, (ii) examine the scope and consistency of associations between peer motivational climate and other psychosocial factors, and (iii) explore its interplay with motivational climates created by other social agents. Three databases were searched and all studies published in English were screened for inclusion. Of the 54 studies included, most were cross-sectional (*N* = 30, 55.55%), and adolescents were the most frequently researched participants (*N* = 42, 77.77%). Peer motivational climate was explored mainly in competitive sports (*N* = 27, 50%), followed by non-competitive sports and exercise (*N* = 21, 38.88%), with only six studies conducted in PE (Physical Education) contexts. Seven research categories and four different interplays among social agents (parents, coaches, teachers, and peers) were identified. Overall, a peer task-involving climate has been more linked to adaptive outcomes while there were also relations between a peer ego-involving climate and maladaptive outcomes (e.g., negative affect and basic psychological need thwarting), but the latter were less consistent. Importantly, the ways peers and adults predicted youth's motivation were distinct. This scoping review can guide not only practitioners into harmonizing different peer motivational climates to optimize youth sports and physical activity experiences but also researchers into future avenues with broader scope and methodologies on peer motivational climate research.

Children's participation in various forms of physical activity involves several interactions with diverse social agents such as coaches, teachers, peers, and parents. Among them, peers' role has become progressively salient in the physical domain. For instance, when youth have a positive peer relationship (friendship and/or accepted by peers), they are more likely to initiate and maintain their physical activity (Jago et al., 2009), experience feelings of competence, enjoyment, self-determined motivation (Smith et al., 2006), and greater self-worth (McDonough and Crocker, 2005). However, peers are cited not only as a reason but also as a barrier to children's sport participation (Howie et al., 2020). Specifically, in their review Howie et al. (2020) stated that youth tend to initiate a sport participation to follow their friends or to make friends in a new place. However, some children who did not join in a youth sport viewed sport as a waste of their free time to be social. Importantly, in the achievement context, peers can serve as markers to enable children to socially compare with others and to judge their competence (Passer, 1996). This capacity is utilized more frequently during the transition into early adolescence when the sources from which they deduce their competence levels shift from adult feedback to peer evaluation and comparison (Horn and Hasbrook, 1986; Horn and Weiss, 1991). Overall, the literature suggests that peers in the sport and physical activity context are gradually becoming significant as children develop with diverse interactions between them and peers.

Motivational climate is one of the contextual variables that explains how social agents in sport and physical activity environment may reinforce or undermine youth's perceptions of competence. Based on achievement goal theory (Nicholls, 1989) and its applications to physical activity (e.g., Duda and Ntoumanis, 2003) an individual's motivation in an achievement setting can be shaped by their achievement goals and perceived contextual motivational climates. An achievement goal is a dispositional factor to interpret one's ability on the basis of individual improvement, skill mastery, and effort (task achievement goal), or on the basis of showing superior ability and outperforming others with less effort (ego achievement goal). Although more recent and nuanced conceptualizations of achievement goals have been tested in the physical activity domain (e.g., Ntoumanis et al., 2009), we cited here the original work by (Nicholls 1989), as task and ego are the type of achievement goals that have been examined in the motivational climate literature. The term motivational climate refers to perceptions of situational motivational cues that induce an achievement goal orientation (Ames, 1992). Two contrasting motivational climates have been proposed by (Ames 1992): a task-involving climate which underscores effort, skills mastery, and individual improvement based on self-referenced criteria, and an ego-involving climate whose emphasis is on social comparison, competition, and outperforming others using normative criteria.

According to studies on motivational climate created by social agents in general, a task-involving motivational climate is predominantly associated with positive psychological and behavioral outcomes such as enjoyment, satisfaction, interest, vigor (see review by Harwood et al., 2015), psychological needs satisfaction, intrinsic motivation, and intention to continue participation in sports (Alvarez et al., 2012). In contrast, it has been reported that an ego-involving motivational climate is more related to negative outcomes such as fear of failure (Gómez-López et al., 2019), burnout (Russell, 2021), and antisocial behaviors (Leo et al., 2015).

By and large, researchers have primarily focused on the motivational climate created by adult social agents, such as coaches, parents, and PE (Physical Education) teachers or overall motivational climate created by both peers and adults simultaneously. Vazou et al. (2005) noted a research gap regarding the potentially impactful but unique role of peer groups in youth sports contexts, leading them to conceptualize “peer motivational climate.” In alignment with an adult-created motivational climate, a peer motivational climate also includes two types: a peer task-involving and a peer ego-involving climate. A task-involving climate is argued to satisfy the three basic psychological needs of competence, autonomy, and relatedness (Ntoumanis and Biddle, 1999). Ntoumanis and Vazou (2005) interview-based analysis identified five dimensions of peer motivational climate: three related to a task-involving climate—Improvement, Relatedness Support, and Effort—and two related to an ego-involving climate—Intra-Team Competition/Ability and Intra-Team Conflict. Notably, “relatedness support” and “intra-team conflict” emerged as unique aspects of peer motivational climate, setting it apart from adult-created climates and underscoring peers‘ distinctive impact on youth athletes' experiences in sports and physical activity.

Currently, 20 years after the concept of peer motivational climate was introduced, no systematic review has comprehensively summarized the literature on motivational climates created by peers in sports and physical activity contexts. While existing reviews have focused on overall motivational climates created by multiple social agents simultaneously (Braithwaite et al., 2011; Harwood et al., 2015; Lacerda et al., 2021; Ntoumanis and Biddle, 1999), peer-specific influences remain less explored. We selected a scoping review framework, defined as “aiming to map rapidly the main concepts of a certain research area, which is complex but has not been reviewed comprehensively” (Mays et al., 2001). Mapping the trajectory and pace of peer motivational climate research can reveal where the field currently stands and guide its future direction. This scoping review seeks to assess the extent of research on peer motivational climate through three specific objectives: (1) identify study characteristics and methodologies used to measure youth perceptions of peer motivational climate in youth, (2) examine the scope and consistency of outcomes that have been investigated with peer motivational climate constructs, and (3) explore the influences of task- and ego-involving climates created by different social agents, alongside peers, on various outcomes.

## Method

This review followed the guidelines of Preferred Reporting Items for Systematic Reviews and Meta-Analyses extension for Scoping Reviews (PRISMA-ScR) (Tricco et al., 2018). Supplementary Table 1 presents the checklists for Scoping Reviews. Specifically, four stages were undertaken: (1) create inclusion/exclusion criteria, (2) identify relevant studies, (3) extract data, (4) Organize the results. The review protocol was developed but not registered; future publication on the Open Science Framework is recommended.

### Inclusion and exclusion criteria

We included all articles on the topic of peer motivational climate in youth sports, physical activity, and PE context for typically developing youth. We also included gray literature that was not officially published in academic journals, such as student theses and dissertations, as they involved the collection of original data from participants. Only articles that were published or accessible online since 2005 were included, as the first concept paper on “peer motivational climate” was published that year. Since the World Health Organization (n.d.) defines the age range of “adolescents” as individuals from 10 to 19 years and “youth” as those from 15 to 24 years, studies that included participants within these age range (10-24 years) were accepted although they also included participants out of the age criteria. However, studies that had large gap in the age range were excluded (i.e., 16-39 years and 16-54 years). Papers not written in English and those without original data (e.g., reviews, books, or research protocols) were excluded.

### Search strategy

**Figure 1:**
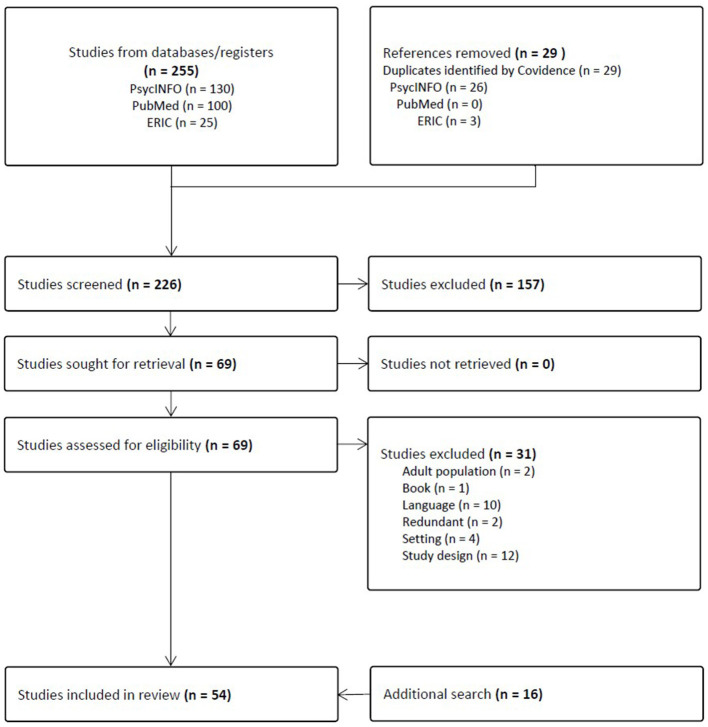
PRISMA flow diagram of the study selection process.

We chose three main databases: PubMed, PsycInfo, and Eric since they are closely related to the field of sport and exercise psychology, and they can broadly provide articles related to youth sport and physical activity. The search process consisted of three steps: First, the first author retrieved studies from three databases using the keywords (peer motivational climate) AND (sports) OR (physical activity) to capture any type of physical activity (e.g., PE or after-school physical activity) while removing duplicates. Second, we conducted a reference search by reviewing all relevant literature cited in the selected articles. Third, an additional hand search was performed using the search engine “Google Scholar,” resulting in identifying 16 additional studies. The PRISMA flow diagram of the study selection process is shown in Figure 1.

### Selection of sources of evidence

Three authors screened titles and abstracts for relevance according to the inclusion and exclusion criteria and resolved discrepancies through discussion. Studies most aligned with the research questions were selected after a full-text review. This entire screening process was conducted using the online platform “Covidence” (Available online at https://www.covidence.org/w). The initial search began in June 2023 to identify articles from the selected databases and was updated through October 2024 with additional searches using search engine and reference lists.

### Data extraction and synthesis

Data was charted using an excel sheet with no specific template. Based on the review objectives, the first author mainly extracted all constructs examined in relation to peer motivational climate and grouped them accordingly. Another author independently reviewed and verified the constructs in each category, and discussed with the first author if there was any disagreement regarding data coding procedure and selection. The overall findings were summarized in one table (Supplementary Table 2) and two figures. Supplementary Table 2 presents various information from each paper, including demographics (authors, country of study, sample size, age, context, and sports level of participants), and study characteristics (purpose of study, study design, outcome measures, theories (in addition to AGT), measurement of peer motivational climate, results, and future recommendation).

## Results

The screening process began with 255 references. After removing duplicates, 226 articles remained. Then, 157 additional studies were excluded through title and abstract screening. Subsequently, the full texts of 69 articles were assessed for eligibility, resulting in the elimination of 31 more articles for various reasons (i.e., adult population, study designs, language, setting, redundancy, and book chapters). In addition to the 38 papers extracted through systematic screening, 16 more articles were added from additional searches. Ultimately, a total of 54 studies were selected for analysis (Figure 1).

### Study characteristics

The most commonly used design was cross-sectional (*N* = 30, 55.55%), followed by longitudinal (*N* = 11, 20.37%), qualitative (*N* = 6, 11.11%), experimental (*N* = 4, 7.4%), and mixed method (*N* = 3, 5.55%). The leading countries of research were the United States (*N* = 12, 22.22%), the United Kingdom (*N* = 12, 22.22%; 5 in England, and 1 in Wales), Canada (*N* = 6, 11.11%), Estonia (*N* = 4, 7.4%), Spain (*N* = 3, 5.55%), Sweden (*N* = 3, 5.55%), Belgium, Turkey, Hong Kong, South Korea, France, and Portugal (*N* = 1 each, 1.85% each)(for full information on study characteristics see Supplementary Table 2). The number of participants ranged from 12 to 2,219, with a median of 244 participants. Adolescents were the focus in 42 studies (77.77%) while adults and children were less frequently studied. Peer motivational climate was examined in competitive sports (*N* = 27, 50%), non-competitive sports and exercise (*N* = 21, 38.88%), and less frequently in PE (*N* = 6, 11.11%). Regarding sports, 24 (44.44%) studies included both individual and team sports, 19 (35.18%) studies included team sports and 2 (3.7%) studies included individual sports.

### Measurement for peer motivational climate

For the measurement of peer motivational climate, 45 (83.33%) of the quantitative studies used the Peer Motivational Climate in Youth Sport Questionnaire (PeerMCYSQ, Ntoumanis and Vazou, 2005). The PeerMCYSQ was developed based on qualitative investigations with youth sport participants conducted by Vazou et al. (2005), and comprises five factors (Improvement, Relatedness support, Effort, Intra-team competition and Intra-team conflict), measured with 21 items. The scale begins with the stem question, “on this team, most athletes”. Improvement refers to encouraging and providing feedback to teammates to support skill development (e.g., “help each other improve”). Relatedness Support refers to fostering feelings of belonging and being part of a group, as well as the creating a friendly team atmosphere (e.g., “make their teammates feel accepted”). Effort refers to emphasizing the importance of exerting effort and trying one's hardest (e.g., “are pleased when their teammates try hard”). Intra-team Competition reflects striving to outperform teammates and engaging in social comparison (e.g., “encourage each other to outplay their teammates”). Intra-team Conflict refers to negative and unsupportive behaviors displayed by teammates (e.g., “make negative comments that put their teammates down”). The PeerMCYSQ is rated on a 7-point Likert scale ranging from 1 (“strongly disagree”) to 7(“strongly agree”). One study developed a shortened version of the PeerMCYSQ (Alcaraz et al., 2013).

Only two studies used alternative questionnaires: The Significant Others' Goal-Involving Roles in Sport Questionnaire (SOGIRSQ, Le Bars et al., 2006) and the Learning and Performance Orientation in Physical Education Classes (LPOPECQ, Papaioannou, 1994). Specifically, The SOGIRSQ was developed to assess athletes' perceptions of task and ego-involving climates created by significant others. It was developed by extracting relevant items from existing questionnaires (i.e., Parent-Initiated Motivational Climate Questionnaire, PIMCQ, White et al., 1992; Perceived Motivational Climate in Sport Questionnaire, PMCSQ-2, Newton et al., 2000), supplemented by qualitative investigations with athletes. The SOGIRSQ consists of six factors measured with 19 items. The three task-involving factors are Promotion of Leaning by the Coach (e.g., “the coach is satisfied when everybody improves”), Promotion of Leaning by Parents (e.g., “my parents follow closely my technical and tactical improvement”), and Pursuit of Leaning by Athletes (e.g., “athletes are very satisfied when they have spent a lot of effort on technical or tactical work”). The three ego-involving factors are Promotion of Comparison by the Coach (e.g., “the coach only looks after those who obtain good results in competition”), Promotion of Comparison by Parents (e.g., “my parents attach great importance to how I place in competition”), and Pursuit of Comparison by Athletes (e.g., athletes are very satisfied when they do better than their training partners”). Responses are recorded on a 5-point Likert scale (1 = “strongly disagree” to 5 = “strongly agree”).

The LPOPECQ (Papaioannou, 1994) was created to assess learning- and performance-oriented climates in physical education classes. The questionnaire was developed through expert evaluation of relevant items from existing questionnaires. It includes four dimensions comprising 22 items: Learning-oriented climates created by peers (five items, e.g., “my colleagues are very satisfied when I learn something new”), Performance-oriented climates created by peers (five items, e.g., “my colleagues try to gain rewards by outperforming me”), Learning-oriented climates created by PE teacher (six items, e.g., “the teacher looks most satisfied when every student learns something new”), and Performance-oriented climates created by PE teacher (six items, e.g., “the teacher looks completely satisfied with those students who manage to win with little effort”). Following the stem question “During physical education…,” items are rated on a 5-point Likert scale (1 = “totally disagree” to 5 = “totally agree”). Finally, one study developed the Individual Sport Motivational Climate questionnaire (ISMC), which assesses peer-, parent-, and coach-created climates, as well as sport reward structure (i.e., perception of the structure of the sport as a whole) in individual sport contexts (Smith, 2010).

### Categories in peer motivational climate research

**Figure 2:**
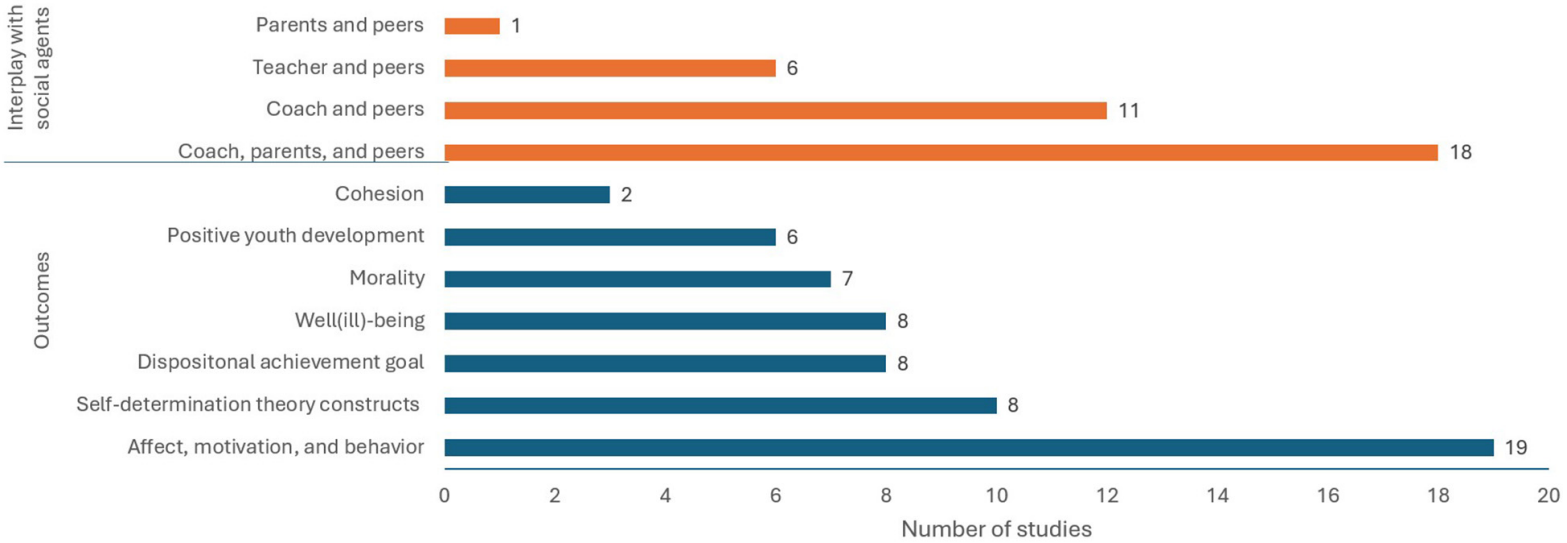
Frequency of studies on scoping review categories.

**Figure 3:**
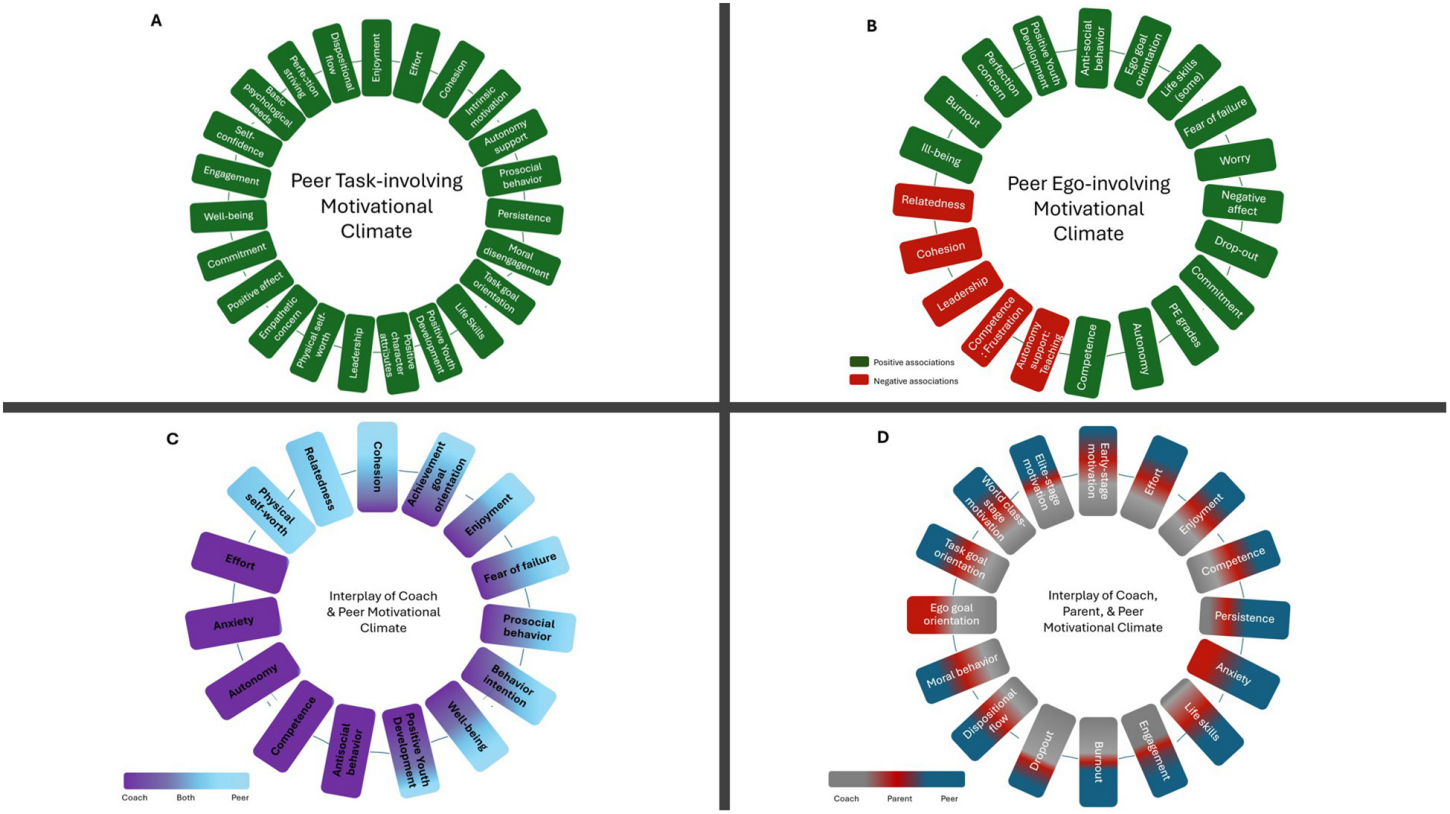
Association of peer task-involving and peer ego-involving motivational climates. **(A)** Associations of peer task-involving climate with outcomes identified in the review. **(B)** Associations of peer ego-involving climate with outcomes identified in the review; **(C)** Interplay between peer and coach motivational climate. **(D)** Interplay among peer, coach, and parental motivational climates; the proportion of each color in the outcome segments represents the relative degree of association with each social agent on **(C, D)**.

Figure 2 presents the number of studies that explored constructs in seven categories and interplays with social agents. The seven categories were identified related to (1) self-determination theory constructs, (2) morality, (3) cohesion, (4) dispositional achievement goal, (5) positive youth development, (6) affect, motivation, and behavior, and (7) well (ill)-being. The social agents investigated in the peer motivational climate literature include coaches, PE teachers, and parents. An overall depiction of the peer task-involving and peer ego-involving motivational climates and their associated outcomes is provided in Figure 3 (panels A and B). Additionally, panel C illustrates the interplay between peer and coach motivational climate, while panel D shows the interplay among peer, coach, and parental motivational climates on predicting various outcomes.

#### Self-determination theory constructs

Eight studies (10, 12, 19, 23, 38, 41, 45, 47; see Supplementary Table 2) explored basic psychological needs, motivation, and autonomy support from coach, and parents, in relation to peer motivational climate. These studies employed cross-sectional, qualitative, and experimental methods. A peer task-involving climate was positively linked to competence, autonomy, and relatedness psychological need satisfaction (Cheon et al., 2019; Rodrigues et al., 2020). Among these needs, relatedness was the strongest correlate to peer motivational climate (Magbanua Jr, 2015). A longitudinal study showed that peer task-involving motivational climate predicted intrinsic motivation over one-year training period (Jõesaar et al., 2012). Other longitudinal studies also reported an indirect effect of peer task-involving motivational climate on intrinsic motivation through the satisfaction of these three needs, while a peer ego-involving motivational climate was negatively associated with relatedness and self-determined motivation (Hein and Jõesaar, 2014; Jõesaar et al., 2011), and positively with competence frustration (Chu, 2018). Other longitudinal studies identified an indirect effect of autonomous motivation between peer task-involving motivational climate and engagement, as well as a mediating effect of controlled motivation between peer ego-involving motivational climate and burnout (Isoard-Gautheur et al., 2021). Notably, peer ego-involving motivational climate had positive associations with competence and autonomy need satisfaction (Rodrigues et al., 2020). Further, peer ego-involving motivational climate showed a positive indirect effect on children's physical education grades through basic psychological needs satisfaction.

#### Morality

Seven studies (13, 22, 23, 25, 41, 44, 49) have examined the links between peer motivational climate, moral behaviors and moral disengagement in youth sports, utilizing cross-sectional, longitudinal, and intervention methods. Peer task-involving motivational climate was found to be positively associated with prosocial behaviors (Cheon et al., 2019; Kavussanu and Ring, 2021; Magbanua Jr, 2015; Ntoumanis et al., 2012), and moral disengagement (Girard, 2020). Additional studies indicated positive associations between peer ego-involving motivational climate and antisocial behaviors (Cheon et al., 2019; Davies et al., 2016; Leo et al., 2015; Ntoumanis et al., 2012).

#### Cohesion

Two studies (35, 48) examined the link between peer motivational climate and team cohesion. In a cross-sectional study, a peer task-involving motivational climate among female adolescent athletes was positively associated with task cohesion, while intra-team conflict in a peer ego-involving motivational climate negatively related to both task and social cohesion (Weiss et al., 2021). A longitudinal study showed that early-season peer ego-involving motivational climate predicted lower mid-season task cohesion, while early-season task cohesion positively predicted mid-season peer task climate (McLaren et al., 2017).

#### Dispositional achievement goal

Considering that the empirical work on the motivational climate created by peers is based on achievement goal theory, it is surprising that only eight studies (5, 7, 9, 21, 27, 32, 36, and 42) explored the relations between peer motivational climate and achievement goal orientations at a dispositional level. Cross-sectional studies found a positive association between a peer task-involving motivational climate and task goal orientation (Atkins et al., 2015; Beck et al., 2017; Ingrell et al., 2019; Smith, 2010), as well as a positive link between peer ego-involving motivational climate and ego goal orientation (Webb and Forrester, 2016). However, some studies showed mixed findings (Vazou, 2010; Warburton, 2017), with one study associating peer task-involving motivational climate with performance goals (Warburton, 2017). The other study did not show significant association between peer motivational climate and achievement goal orientation (Le Bars et al., 2009).

#### Positive youth development

Six studies (20, 24, 28, 33, 37, 46) explored the relations between peer motivational climate and positive youth development (PYD) constructs, including, developmental experiences, positive character attributes, life skills, perfectionism, and self-concept. One study focused on underserved girls, showing that a peer task-involving motivational climate was positively associated with positive developmental experience, especially for girls from non-migration backgrounds, but interestingly, a peer ego-involving motivational climate also showed a positive relation with positive developmental experience (Schaillée et al., 2017). Another study on high school athletes found that peer task-involving motivational climate was linked to positive character attributes (Agans et al., 2018). Interestingly, peer task-involving motivational climate was positively associated with all eight life skills, but also peer ego-involving motivational climate was positively associated with some life skills like teamwork, and goal setting (Mossman et al., 2021). Peer task-involving motivational climate was positively related to perfectionistic striving and peer ego-involving motivational climate was positively linked to perfectionistic concerns (Greblo et al., 2016). Lastly, an experimental study implementing a leadership program for female varsity athletes noted an increase in peer task-involving motivational climate and a decrease in peer ego-involving motivational climate (Duguay et al., 2016). For the self-concept construct, there was not a significant association with peer motivational climate (Taylor et al., 2014).

#### Affect, motivation, and behavior

Affective, motivational, and behavioral outcomes were investigated with youth athletes' peer motivational climate in 19 papers (3, 5, 9, 10, 11, 13, 14, 15, 17, 20, 21, 26, 29, 30, 31, 38, 39, 40, 54). A peer task-involving motivational climate was positively associated with physical self-worth, enjoyment (Vazou et al., 2006, indirectly through task goal orientation, Atkins et al., 2015), self-esteem (through task goal orientation, Atkins et al., 2015), empathic concern (Ettekal et al., 2016), effort (Chan et al., 2012; Mellano, 2019), engagement (Mellano, 2019), commitment (Tamminen et al., 2016), positive affect (Webb, 2013), self-confidence (Urquhart, 2016), persistence (directly; Jõesaar and Hein, 2011; Le Bars et al., 2009, as well as through satisfaction of the three basic psychological needs; Jõesaar et al., 2011), intention to continue the sport (Mellano, 2019, through task goal orientation, and competence, Atkins et al., 2015), intention to return to the team (McLaren et al., 2023), and physical activity behavior (Taylor et al., 2014). In contrast, a peer ego-involving motivational climate was positively related to fear of failure (Gómez-López et al., 2019), worry (Ingrell et al., 2016), negative affect (Webb, 2013), and dropout (Chu, 2018). Interestingly, peer ego-involving motivational climate was also positively linked to some adaptive outcomes, including commitment (Tamminen et al., 2016), and competence (directly; Chan et al., 2012; Smith, 2010, indirectly through task goal orientation, Atkins et al., 2015). Moreover, more experienced players reported perceiving higher levels of peer ego-involving motivational climate (Gómez-López et al., 2019). However, a few studies did not show significant associations between peer motivational climate and affective, motivational, and behavioral outcomes (Atkins et al., 2013; Ntoumanis et al., 2012).

#### Well (ill)-Being

Eight studies (6, 13, 38, 40, 43, 47, 53, 54) explored how psychological wellbeing is related to their perceptions on peers in sports and physical activity contexts. Consistently, a peer task-involving motivational climate was positively associated with wellbeing (Chu, 2018; Habeeb et al., 2023; McLaren et al., 2023; Mellano, 2019; Ntoumanis et al., 2012), while a peer ego-involving motivational climate was associated with ill-being, including burnout (Chu, 2018; Habeeb et al., 2023; Ntoumanis et al., 2012; Smith et al., 2010). A longitudinal study also found that peer task-involving motivational climate predicted wellbeing through autonomous motivation, while peer ego-involving motivational climate indirectly predicted ill-being via controlled motivation (Isoard-Gautheur et al., 2021). Interestingly, female athletes reported high levels of peer task-involving motivational climate and low levels of peer ego-involving motivational climate but perceived high training load and stress (Smith et al., 2010). The other study did not show a significant association between peer motivational climate and internalizing features (Dandan, 2019)

### Interplays with other social agents

#### Influence of coach and peer-created motivational climates

Eleven studies (3, 7, 12, 13, 16, 23, 31, 33, 39, 40, 48) examined the influence of coaches and peers, using different outcome variables (Figure 3C). In the research investigating the influence of such climates on youth athletes' affective and behavior outcomes, the results showed that peer task-involving motivational climate was the only correlate of physical self-worth, both coach and peer task-involving motivational climate were positively associated with enjoyment (Vazou et al., 2006) and intention to continue (Mellano, 2019), but only coach ego-involving motivational climate had ties with athletes' anxiety (Vazou et al., 2006). Another study reported that the relationships between athletes' dispositional goals and the influence of coaches and peers varied across players and contexts (Vazou, 2010). Also, similar results appeared when examining the impact of coaches and peers on moral attitude, wellbeing, and behavioral intention (Ntoumanis et al., 2012). Further, another study explored how coach and peer motivational climates are associated with basic psychological needs and moral behavior of youth athletes (Magbanua Jr, 2015). It was reported that a peer task-involving motivational climate was positively related only to relatedness need and prosocial behavior whereas a coach task-involving motivational climate had a positive association with autonomy and competence needs and a coach ego-involving motivational climate had ties to antisocial behaviors.

Autonomy support from social agents played a critical role in children's physical activity. For instance, a longitudinal study reported that both autonomy support from coach and peer task-involving motivational climate predicted intrinsic motivation over 1 year of training (Jõesaar et al., 2012). Interestingly, however, coach autonomy support predicted a year-later peer task-involving motivational climate, but the reverse effect was not found. When exploring positive youth development, the researchers found that the coach showed stronger association than peers, although both coach and peers positively associated with positive youth development (Schaillée et al., 2017). In the study examining fear of failure, it was reported that both team (coach and peers) and peer ego-involving motivational climate were positively related to athletes' fear of failure (Gómez-López et al., 2019). One study investigating cohesions showed that both team (coach and peers) and peers had positive associations with cohesion, and only peer ego-involving motivational climate played a negative role in cohesion (Weiss et al., 2021). The last article examining the motivational climate created by team (coach and peers), team captain, and peers reported only team captain task climate was positively associated with enjoyment (Urquhart, 2016). The other study developed and validated the short form of peer motivational climate and coach autonomy support scale (Alcaraz et al., 2013).

#### Influence of teacher and peer-created motivational climates

Six studies (20, 32, 41, 45, 50, 51) explored the roles of PE teachers and peers in the school context. In a longitudinal study looking at dispositional achievement goals, the results showed mixed associations between goal orientations and motivational climates created by teachers and peers (Warburton, 2017). Furthermore, the only association found was between peer ego-involving motivational climate and PE grades via needs satisfaction while there was no link between teacher ego-involving motivational climate and PE grade (Rodrigues et al., 2020). However, when a teacher task-involving motivational climate increased over time, a peer ego-involving motivational climate declined (Warburton, 2017). Also, an experimental study at school reported that autonomy supportive teaching enhanced peer task-involving motivational climate and diminished peer ego-involving motivational climate (Cheon et al., 2019). The study explored children's self-concept and physical activity in the transition from primary to secondary school and delineated that the teacher's impact was stronger than peers' in this transition period (Taylor et al., 2014). Also, in an experimental study where PE teachers implemented a morality-focused pedagogical model for students in secondary schools, a peer task-involving motivational climate increased and peer ego-involving motivational climate decreased (Rillo-Albert et al., 2021). Lastly, in the typical PE context, girls reported high teacher and peer ego-involving motivational climate (Tidmarsh et al., 2022).

#### Influence of parents and peer-created motivational climates

We found only one study (15) focused on the interplay between parents and peers. The study, examining how the influences of parents and peers aged from 10 to 14 are related to affect and behavior, reported that only parental task-involving motivational climate predicted self-esteem, competence, and enjoyment, which in turn led to intention to continue their sports, while no significant effects were found for peer motivational climate (Atkins et al., 2013).

#### Influence of parents, coach, and peer-created motivational climates

Eighteen studies (4, 5, 8, 9, 11, 14, 18, 19, 21, 22, 25, 34, 36, 38, 42, 46, 52, 53) examined the interplays between parents, coaches, and peers in youth sports and physical activity, revealing distinct influences based on context and outcomes. Although most studies were cross-sectional and presented mixed results, many of them showed distinct influences of peers. In one study, task climates from all three agents were positively associated with task goal orientations, with parents and peers exerting the strongest influence (Atkins et al., 2015). Another study found that only peer task-involving motivational climate was related to task orientation, while coach ego-involving motivational climate aligned with ego orientation (Ingrell et al., 2019). Additionally, for individual youth athletes only motivational climates created by coach and peers were associated with task orientation and only adults-created motivational climates (coach and parents) were related to ego orientation (Smith, 2010). In contrast, a separate study reported that teams' (coach and peers) and parents' task and ego-involving motivational climates were positively associated with task and ego orientation, respectively, with no associations for peer motivational climate (Beck et al., 2017). In studies on youth athletes' moral behaviors, parents had the strongest impact on moral behaviors (Davies et al., 2016; Leo et al., 2015), though coaches and peers also played a role (Leo et al., 2015), with teams (coaches and peers) being more influential for younger athletes, and peers for older (Davies et al., 2016).

In terms of effort, enjoyment, and competence, mothers influenced children more than fathers, coaches and peers, and peers and fathers had a stronger impact on adolescents and coaches were influential on both age groups (Chan et al., 2012). Autonomy support from parents was found to be stronger on athletes' motivation than that from coaches, though coaches' support was more crucial for shaping peer motivational climates than that of parents (Hein and Jõesaar, 2014). Regarding life skills, peer task-involving motivational climate was the strongest positive correlate, and parental ego-involving motivational climate the strongest negative (Mossman et al., 2021). For athlete's burnout, dropout and engagement, coaches (Chu, 2018) as well as coaches and peers (Habeeb et al., 2023) played a more substantial role than parents. A cross-sectional study showed that task-involving motivational climates from all three social agents positively correlated with dispositional flow (Çaglar et al., 2017). In addition, the study with individual youth athletes also found that the perceived competence was associated with all three social agents (Smith, 2010). In longitudinal studies, athletes who continued their sport experienced more autonomy support from parents and a peer task-involving motivational climate than those who dropped out (Jõesaar and Hein, 2011), whereas task-involving motivational climates of all three social agents positively predicted sports persistence, with peers exerting the strongest influence (Le Bars et al., 2009).

Qualitative studies revealed that peer influence was distinct from that of coaches and parents (Keegan et al., 2009), although the roles of coaches and parents were similar in motivating elite athletes (Keegan et al., 2010). In a study of national and world-class athletes, overlapping influences from coaches and peers were noted (Keegan et al., 2014). Finally, research on elite adolescent football players found that as athletic development progressed, the roles of coach and peers became more significant, while parental roles and influence diminished (McCann et al., 2022).

## Discussion

This scoping review aimed to comprehensively scope the literature on peer motivational climate in the several physical activity contexts. Unlike existing reviews on adult-created motivational climates, this study is the first to summarize the evidence on how peers contribute to the motivational climate framework. We believe it provides a valuable bi-decennial overview of the topic and highlights future directions by identifying gaps in the existing literature.

### Methodology

The analysis of study methodology revealed that over half of the studies employed cross-sectional correlational designs. While fewer in number, studies that utilized stronger methods, like longitudinal and intervention designs, were identified. Cross-sectional studies can identify associations between peer motivational climate and variables of interest but limit causal inferences. For example, a cross-sectional study indicated that coach and peer task-involving motivational climates were more associated with task cohesion than social cohesion (Weiss et al., 2021) but it could not determine the direction of this relationship. In contrast, a longitudinal study showed that peer ego-involving motivational climate negatively predicted mid-season cohesion (McLaren et al., 2017), providing stronger insights due to measurements taken at multiple time points, however, causality can again not be established with such designs.

Utilizing more advanced methods such as longitudinal design is particularly important to understand the temporal formation of peer motivational climate. For instance, in youth sport teams it is likely that the interactions between athletes are less impactful as some new players join the team at the beginning of the season. However, as the season or session progresses, their interactions can become more direct and at least stronger than those at the beginning of the season although the valence (positive or negative influence) may vary (if the same coach remains). In the same logic, how peers communicate in the mid-season can also differ how they do at the end of the season. Thus, tracing peer motivational climate across time will provide a developmental perspective of the peer- motivational climate for practitioners.

Studies examining peer motivational climate as an outcome were conducted in sports (Duguay et al., 2016), physical education (Cheon et al., 2019; Rillo-Albert et al., 2021), and after-school settings (Dandan, 2019). Outside of this review, some interventions reinforced children's achievement goals through adult stakeholders (Bortoli et al., 2015; McLaren et al., 2015; Mouratidou et al., 2022), primarily focusing on adult-athlete or student relationships. Future research should develop interventions that specifically target peer interactions within the motivational climate framework, as evidence from this review indicated that peer motivational climate strongly predicts adaptive outcomes in youth sport and physical activity. Interventions could draw on theoretical frameworks such as Achievement goal theory and Self-determination theory) to test the effects of peer task- and ego-involving motivational climate using confederates who model each climate in the exercise and physical activity settings. Additional studies could examine the combined effects of peer motivational climate and basic psychological need support (e.g., autonomy, relatedness) to clarify how peer influences operate across theoretical perspectives.

#### Study characteristics: age and experience

The results indicated that adolescent athletes were the most frequently studied group, followed by adults and children. Most studies focused on participants aged 12 and older, an important developmental stage where children begin to differentiate between ability and effort in evaluating their performances (Roberts, 1993). As they mature, their criteria for success shift from parental feedback to peer evaluations (Horn and Hasbrook, 1986; Horn and Weiss, 1991). This suggests that even within the same adolescent phase, athletes aged 14 may perceive social comparison differently than their 18 year old counterparts. For example, research on peer and coach motivational climates showed that male and older athletes reported a more peer-ego involving motivational climate (Vazou, 2010).

Furthermore, it is important to examine how children's thought processes become more complex as they mature. For example, from the perspective of achievement goal theory, early adolescents might distinguish only between task- and ego-involving motivational climate while older adolescents are more likely to interpret these climates in a more complex way, by differentiating between approach ego (demonstrating higher competencies over others) and avoidance ego (avoiding demonstrating incompetencies relative to others) (Barkoukis et al., 2007; Elliot, 1997). However, many studies in this review did not consider age in their analysis, and while some included years of training, this factor is also crucial as young athlete's perceptions of peers evolve. To understand the level of complexity at various developmental stages, future research should target specific age groups and control for years of being in a team with corresponding methodological and theoretical approaches.

#### Study characteristics: types of sport

An important consideration regarding study characteristics is that most studies included participants from both individual and team sports, yet few studies explicitly examined sport types as a contextual factor in their analysis. This may reflect the considerable variability that exists both within and across sports, as peer interactions can differ substantially depending on the structure, culture, and training environment of a given sport, rather than sport type alone. For example, although team sports such as soccer or baseball often involve coordinated efforts toward a shared competitive goal the extent and quality of peer interaction can vary greatly across teams. Similarly, individual sports such as golf or gymnastics may involve different levels of cooperation, comparison, and peer support depending on training structure and competitive demands. Thus, categorizing sports strictly “individual” or “team” may oversimplify the complex social dynamics experienced by youth athletes.

One study (Smith et al., 2010) found that higher perceptions of peer task-involving motivational climate and lower intra-team conflict were associated with burnout only in individual sports. Rather than reflecting inherent differences between individual and team sports, this finding may point to contextual differences in how peer interactions are structured and perceived across sport settings. From the achievement goal perspective, peer motivational cues may be more salient in contexts where peer interaction is less frequent or explicit. T Similarly, from a self-determination theory perspective, athletes in different sport contexts may experience varying opportunities for autonomy and relatedness support depending on training structure, rather than sport type *per se*. To advance understanding of youth athletes' perceptions of peer motivational climate, future research should move beyond broad sport classifications and consider more nuanced contextual factors, such as training organization, competitive structure, and peer interdependence. Aligning measurement tools with these contextual characteristics may provide a more precise understanding of peer dynamics across youth sports and physical activity settings (Evans et al., 2012).

#### Study characteristics: context

Most studies focused on sports, with few studies examining non-competitive contexts like school or gyms. Peer relationships are likely to differ across settings. The findings (Cheon et al., 2019; Rodrigues et al., 2020; Rillo-Albert et al., 2021; Taylor et al., 2014; Tidmarsh et al., 2022; Warburton, 2017) suggest that, in PE contexts, teachers may have a more significant impact on children's physical activity experiences than peers, who might lack motivation to engage in PE. Nevertheless, positive teaching styles can enhance children's positive peer-to-peer interactions and peer motivational climate. Similarly, in exercise settings such as fitness centers, peer task-involving motivational climate can play an important role in influencing motivation and supporting the satisfaction of the basic psychological needs (Murcia et al., 2008).

Importantly, when context changes, the tool used to assess peer motivational climate should be adapted to reflect what is meaningful to the specific population. The focus of contextual adaptation should be on identifying the psychological needs that individuals in that setting seek to satisfy. The original PMCYSQ was primarily grounded in achievement goal theory, emphasizing competence-based criteria for success. However, in a non-competitive exercise context, individuals may be more concerned with autonomy (the degree of freedom they experience), relatedness (how welcomed and accepted they feel by others), or evaluate their competence based on self-referenced improvement and learning rather than normative success (e.g., winning, which may not be relevant in such settings). Therefore, adapting the scale to fit the specific context and aligning it with an appropriate theoretical framework can enhance our understanding of the unique characteristics of that environment. For instance, a scale designed for a non-competitive exercise and physical activity setting might include an Autonomy Support factor, which is not included in the original PMCYSQ, assessing the extent to which participants feel supported by their peers in expressing their preferences and making activity-related choices. Future research should explore the specific needs of this population within respective contexts (e.g., identifying the types of peer interactions that are most common or valued). Expanding such research to include everyday physical activities or exercise groups across diverse age ranges could help uncover the peer interaction patterns that are most prevalent in each context.

Gender is an important consideration in the peer motivational climate research, as several studies observed gender-based differences in perceptions of peer motivational climate. In youth sports, females have reported perceiving a higher peer task-involving motivational climate (Smith et al., 2010) and a lower peer ego-involving motivational climate than males (Vazou, 2010; Webb and Forrester, 2016). Tidmarsh et al. (2022) found that only girls perceived both teacher and peer ego-involving motivational climate in PE classes. Although it is premature to link gender differences in perceptions of peer motivational climate directly to specific contexts (e.g., sport or school), consistent evidence suggests distinct perceptions between males and females in motivational climate created by peer groups. Examining gender differences is particularly important because perceptions of motivational climate influence how individuals contribute to its creation. Also, interactions between same- or mixed- gender groups within the same motivational climate (e.g., male with male, or male with female) may vary depending on their preferences and social dynamics. Therefore, exploring gender effects and contextual associations through adaptive methodological approaches could provide deeper insights into these mechanisms.

### The scope of outcomes associated with peer motivational climate

This scoping review identified diverse outcome variables reflecting the multifaceted impact of peer motivational climate across contexts. Among these, affective and behavioral outcomes emerged as particularly prominent, highlighting the central role of emotions, enjoyment, and intention to continue or dropout in understanding how peers influence motivation and behavior. Notably, relatively few studies have examined behavioral intentions over time, underscoring the need for longitudinal approaches to capture how peer-related motivational processes evolve and contribute to sustained engagement or dropout. Specifically, longitudinal studies reported that a peer task-involving motivational climate positively predicted persistence (Jõesaar et al., 2011; Le Bars et al., 2009) while another study found no significant relationships between peer motivational climate and intention to continue (Ntoumanis et al., 2012). Due to the inconsistent findings, future research should examine behavioral outcomes with various mediating factors from a longitudinal perspective.

As the scoping review showed, cohesion was less frequently studied despite its importance in group dynamics (Eys and Brawley, 2018). Diverse environmental factors can make significant contributions to continuous team development (Arrow et al., 2004). Since peer interactions differ from those with adult social agents, how athletes perceive their roles within the team may vary based on different peer motivational climates. For instance, applying social identity theory (Tajfel and Turner, 1979), athletes may establish an in-group identity for their team while resisting out-group perceptions of other teams. It is anticipated that athletes who perceive their own group as attractive might promote ideal level of peer motivational climate whereas those whose perception of group identity is weak might foster minimal level or any type of peer motivational climate regardless of the norm. Exploring how contrasting peer motivational climates influence these in- and out-group dynamics could provide valuable insights. Likewise, investigating other group development concepts, such as role perceptions or subgroups, may offer intriguing avenues for future peer motivational climate research.

### Inconsistency of outcomes associated with peer motivational climate

A peer task-involving motivational climate is frequently linked to most of the adaptive outcomes across the emerging categories. In contrast, while peer ego-involving motivational climate tends to show positive associations with maladaptive outcomes, the results are less consistent across categories.

Regarding motivation and psychological needs, most of the results showed that a peer task-involving motivational climate was positively associated with the satisfaction of the three basic psychological needs, intrinsic motivation, and autonomy support from coaches and teachers. Notably, satisfaction of the need for relatedness was the most closely associated with a peer task-involving motivational climate (Magbanua Jr, 2015). This contrasts with earlier research indicating that coach task-involving motivational climate was more strongly associated with autonomy (Reinboth and Duda, 2006) and competence (Ahmadi et al., 2012) than relatedness need satisfaction. These associations between task-involving motivational climates from peers and coaches and need satisfaction may stem from the different levels of authority between peers and coaches, which influence how athletes perceive and interpret similar motivational climates.

Peer ego-involving motivational climate has shown greater inconsistencies across outcomes. For instance, Vazou (2010) found that athletes' perceptions of peer ego climate varied significantly based on their achievement ego goal orientations. Moreover, a study conducted in the PE context found that peer ego-involving motivational climate, similarly to peer task-involving motivational climate, predicted both mastery-approach and mastery-avoidance adoption (Warburton, 2017). Other studies have reported positive associations between peer ego-involving motivational climate and outcomes such as life skills (Mossman et al., 2021), commitment, and perceived competence (Chan et al., 2012; Tamminen et al., 2016). Importantly, however, a greater number of null findings has been reported for peer ego-involving motivational climate compared to peer task-involving motivational climate.

These discrepancies should be interpreted with caution. Although some studies report positive associations between peer ego-involving motivational climate and psychological or behavioral outcomes, the presence of negative and null findings precludes drawing firm conclusions. Importantly, these findings should not be interpreted as evidence supporting the intentional promotion of ego-involving motivational climates in youth sport or physical activity contexts. Rather, future research is needed to clarify the conditions under which such perceptions emerge and how they are experienced by youth. In particular, using alternative measurement approaches (e.g., distinguishing approach- and avoidance-oriented ego climate dimensions; Barkoukis et al., 2007), as well as examining moderating factors such as age, sport culture, and type of sport, may help elucidate patterns of peer ego-involving motivational climate perceptions among children and adolescents.

### Interplays with social agents

In addition to studies that focus solely on peer motivational climate in the physical domain, this scoping review identified four types of interplay between motivational climates created by different social agents, with the interaction between coach, parental, and peer motivational climate being the most frequently examined. Results indicate that coaches, parents, and peers have uniquely contributed to youth athletes' sports experiences.

Interestingly, the task-involving motivational climates created by parents and peers often had a more significant effect on athlete's task goal orientations and persistence than the task-involving motivational climate created by coaches (Atkins et al., 2015; Jõesaar and Hein, 2011). Similarly, coach and peer task-involving motivational climates were found to be more influential than parental task-involving motivational climate in predicting athletes' burnout and engagement (Habeeb et al., 2023). The importance of coaches and peers increased as athletes progressed in their athletic development (McCann et al., 2022). This may reflect the different stages of youth athletic development (e.g., sampling, specializing, and investment stages) or the athlete's history in a team (just joining a team vs. playing for a team relatively longer than other members), that may be impacted differently by each stakeholder. As athletes progress in their sport and invest more time and effort, they tend to place greater emphasis on skills, performance, and match outcomes—areas where coaches and peers exert a stronger influence than parents, whose role becomes more limited. This shift may also suggest that certain aspects of parental involvement in athletes' motivation evolve alongside developmental changes. Future research should focus on contextual or dispositional factors such as coaching philosophies (or parenting styles) and the cues used by social agents, to better understand their influence on youth sport experience.

Distinct roles of social agents were observed at different developmental stages regarding predicting moral behaviors. Davies et al. (2016) found that overall teams (coaches and peers) were more crucial for younger athletes, while peers independently played a larger role for older athletes and parents were important across all age groups. Chan et al. (2012) also noted that parents had a significant impact only on children, peers' role was greater for adolescents, and athletes at all ages were influenced by coaches. Although this is a small number of studies, these results align with developmental literature indicating that children increasingly rely on peer feedback or comparison to assess their competence as they transition from late childhood to adolescence (Horn and Hasbrook, 1986; Horn and Weiss, 1991). However, mixed results emerged regarding the influence of adult social agents (coaches and parents) across developmental stages, possibly due to different educational styles or the context in which these agents operate. More research is needed to clarify the dynamics between youth sports participants and adult social agents.

The role of a teacher (e.g., in PE) in the interplay of peer motivational climate with the climate created by other social agents seems understudied in the existing literature. This is surprising, given that positive PE experiences not only enable children to stay active on and off the school (Barkoukis et al., 2010; Cox et al., 2008) but also contribute to enhanced academic achievement (Centers for Disease Control and Prevention, 2010). While not many, the studies in the current review support the positive influences (autonomy support and task-involving motivational climates) created by teachers and peers (Cheon et al., 2019; Rodrigues et al., 2020; Rillo-Albert et al., 2021; Warburton, 2017). Thus, both in-school and out of school physical activity practices should be explored based on how peers interact with one another from the lenses of motivational climate and successfully develop translational research in this area. An example of applying a peer motivational climate to provide enjoyable and need-supportive lessons to students is the field experimental study by Vazou et al. (2019), which manipulated the content of fitness activities in PE by providing positive peer interactions (peer task-involving climate through teamwork and play) compared to negative peer interactions (peer task-involving climate through competitive tasks and structures).

In this scoping review one study explored the interplays between peers, coaches, and the team captain, revealing that only team captain task-involving motivational climate had a positive association with enjoyment (Urquhart, 2016). Although research on the motivational climate created by team captains is limited, this result is not surprising. This may be largely due to the unique position of team captain, in which she or he is able to not only have close communication but also set an expectation of how the behavior of the rest of the athletes should look. When these expectations align with an emphasis on skill mastery and self-improvement, rather than competition against others, athletes experience greater enjoyment in the sports setting. Exploring the distinct roles of peers in exercise contexts (e.g., leaders vs. followers) and how they are intertwined with adult-created motivational climates, would be a valuable direction for future research.

### Limitation and future direction

This scoping review revealed several discussion points but also had limitations. First, while it included a broad array of studies (from primary papers to gray literature) from multiple continents, only those written in English were reviewed. This language limitation restricts the range of key findings that could help us better understand peer motivational climate. To address this, future research can include studies published in non-English journal. Second, this review did not explore the specific tools used to measure peer motivational climate. Although most cross-sectional studies used the PeerMCYSQ (Ntoumanis and Vazou, 2005), as the first tool developed to measure this construct, a small number of studies used distinct tools (Le Bars et al., 2006; Papaioannou, 1994). Importantly, the conceptualization of peer motivational climate may vary across measures. For example, in the PeerMCYSQ (Ntoumanis and Vazou, 2005), Intra-Team Conflict is treated as a defining feature of the peer climate and captures behaviors that actively undermine peer relatedness (i.e., relatedness-thwarting interactions), whereas in other motivational climate frameworks such behaviors are typically conceptualized as consequences of an ego-involving climate rather than as components of the climate itself. As the number of studies that use different tools increases, future research should consider how differences in measurement, validation, and contextual application may influence interpretations of peer motivational climate. Lastly, although this scoping review provided a broad overview of research on peer motivational climate, it did not assess study quality. As more research accumulates, a systematic review and meta-analysis could provide more insightful interpretations of the extant literature.

### Conclusion

Despite the limitations, this scoping review makes a significant contribution as the first to summarize the research evidence on peer motivational climates in sport and physical activity. In summary, our findings indicate that peers play a substantial role in shaping children's experiences in these environments, with various relationships observed between peer motivational climate and a range of outcomes. We highlight the importance of future research using advanced designs and considering factors closely tied to peer dynamics in broader contexts, as well as successfully translating research into practice. Importantly, we hope that adult stakeholders apply these insights in practice to enhance young people's experiences in sport and physical activity. Specifically, adult stakeholders such as coaches, PE teachers and trainers should carefully consider contextual factors such as how their instructional strategies shape peer interactions by avoiding excessive emphasis on peer comparison and minimizing competitive structures (e.g. group formations) that may shift attention toward others' performance (Vazou et al., 2019). Moreover, clarifying the inconsistent effects of peer ego-involving motivational climate and its differential impact relative to adult-created climates remains an important research priority. From an applied perspective, adult stakeholders should prioritize the cultivation of task-involving motivational climates while being attentive to how competitive elements naturally arise within peer interactions. Emphasizing the quality and consistency of task-involving cues may help ensure that such elements do not undermine adaptive motivational experiences for participants.

## Data Availability

The original contributions presented in the study are included in the article/Supplementary material, further inquiries can be directed to the corresponding author.
