# Supplementary material for: A systematic scoping review of peer motivational climate in youth sports and physical activity: a bi-decennial update
Source: Front Psychol. 2026 Mar 11;17:1635666. doi: 10.3389/fpsyg.2026.1635666 (PMC13013351; doi:10.3389/fpsyg.2026.1635666)
Supplement: Supplementary file 2 [file Data_Sheet_2.pdf]

Literature Review of Peer Motivational Climate

ID	Author	Country	Purpose	Sample N	Population (Age)	Context	Ability	Design	Outcome Measures	Theory	Results	Future Recommendation
1	Vazou et al. (2005)	UK(England)	explore peer MC	30 (F-16, M-14)	Teens (12-16 Y)	Individual & Team Sport Clubs and schools	Youth Sport	Qualitative	Peer MC	AGT (Achievement Goal Theory)	PeerMCYS Themes Development	1. Establishment of PeerMCYSQ.<br>2. Interplay of coach - peer MC.<br>3. Peer influence of achievement and competence beliefs
2	Ntoumanis & Vazou (2005)	UK(England)	develop peer MCYSQ	S1: 431 (F-151, M-280)<br>S2: 606 (F-257, M-349)<br>S3: 493 (F-124, M-367)	Teens (11-16 Y)	Individual & Team Sport Clubs and schools	Youth Sport	Cross-sectional ; Measurement Development	PeerMCYSQ	AGT; SDT (Self-Determination Theory)	PeerMCYSQ Development	1. Autonomy support & competence evaluation.<br>2. variations in newly formed teams.
3	Vazou et al. (2006)	UK(England)	Compare coach and peer MC in youth sport on motivational outcomes	493 (F-124, M-369)	Teens (12-17Y)	Individual & Team Sport Clubs and schools	Youth Sport (2 months - 14 years)	Cross-sectional	Physical self-worth, enjoyment, effort, competitive trait anxiety	AGT	1. Physical self-worth (+) = only Peer Task MC;<br>2. Enjoyment (+) = both Peer and Coach Task MC;<br>3. Competitive trait anxiety (+) = only Coach Ego MC;<br>4. Effort (+) = only Coach Task MC	1. Independent & interplay of Coach - Peer MC over time.<br>2. Interplay of parents, coach, peers.<br>3. Variations in perceptions within and between teams.
4	Keegan et al. (2009)	N/A	Explore motivational construct of three social agents and compare with existing themes	40 (F-19, M-21)	Children (7-11Y)	Individual & Team Sports	Youth Sport (beginners - 3 years)	Qualitative	The motivational climates by 3 social agents	AGT	1. Several themes from 3 social agents were similar to existing themes.<br>2. Coaches and parents have a stronger influence on motivation.<br>3. Peer influence on motivation was qualitatively distinctive from adults agents and less consistent.	1. Broader measure is develop and see contradictory behaviors between three social agents.
5	Le Bars et al. (2009)	France	To examine the difference bewteen persistent and dropout athletes in social agents' MCs and it's temporal variations.	1: 104 (F-40, M-64)<br>2. 82 (F-37, M-45)	1. Mean- 17.9Y<br>2. Mean- 16.7Y	Individual sports	Elite	Longitudinal	Significant Other's Goal-Involving Roles in Sport; Perception of success; Physical self-perception; competence; intention of giving up	AGT	1. Task climate by 3 social agents (peer strongest) =(+) persistence. Task orientation +(ass) persistence and no gender difference.<br>2. Coach ego climate increased, task climate by 3 agents decreased over time.	1. Other types of sports.<br>2. More reliable instruments for parents' perception.<br>3. The process of dropout over time.
6	Smith et al. (2010)	Sweden	Associations between Peer motivational climate and burnout of adolesent athletes	206 (F-35.4%,M-64.6%)	Adolescent(16-19Y)	Team and Individual sports	Competitive athletes	Cross-sectional	Perceived stress, burnout	AGT	1. Peer task MC was (-), and Peer ego MC was (+) associated with the burnout perceptions.<br>2. Stress (+) = burnout perceptions.<br>3. Females perceived more training load, stress, and Peer task MC, and less Peer ego MC than males.	1. Multifarious assessmnt (biological markers).<br>2. Longitudinal study examining the origins and process of burnout.<br>3. Gender difference.
7	Vazou (2010)	UK	Examine the variations of peer and coach motivational climats (within and between teams, individual and group level)	483 (F-124, M-359)	Teens (12-17Y)	Team and individual sports from school, club and county	Youth sport(2month - 14years)	Cross-sectional	Achievement goal orienation, Success	AGT	1. Perceptions of peer and coach MC varied considerably between and within team.<br>2. Dispositional achievement goals predicted coach and peer MC differently.<br>3. Boys and older perceived high peer Ego MC and low coach Task MC than girls and younger athletes.	1. Variations on more teams from various sports.<br>2. Individual vs team sports.<br>3. Group or individual-referent item stems.
8	Keegan et al. (2010)	U.K	Explore the MCs of 3 social agents and influence on motivation	79 (F-36, M-43)	Youth (9-18Y)	26 sports	Specializing (Competitive)	Qualitative	Constructions of motivational climates created by three social agents	AGT; SDT	1. Coach, parents, and peers have specific roles for athletes motivation.<br>2. Some of coach's and parents' roles are similar.	1. Enhancing educational programs based on the themes and findings.
9	Smith (2010)	U.K.	develop measurement for individual sports; examine associations between motivational climate (coach, parents, and peers), and goal orientation, and competence	Study2: Qual-12, Quan-292 (F-148, M-144)<br>Study 3a: 423 (F-215, M-208),<br>Study 3b: 273 (F-131, M-142),<br>Study 4: 186 (F-75, M-109)	Study 2: qual (13-17Y), quan (Mean-15),<br>Study 3a: 14-23Y,<br>Study 3b: 15-26Y,<br>Study 4: 16-19Y	Individual sports	Junior national and club atheltes	Quantatative and qualitative; Measurement Development	Coach, parents and peer MC, achievement goal orientation, and competence.	AGT	Development of ISMCQ (Individual Sport Motivational Climate Questionnaire);<br>1. Only coach and peers (ass)task orientation, adults (coach and parents) (ass) ego orientation.<br>2. Before considering importance of others, adults (ass) more competence, but after considering it, peers (ass) more competence.<br>*(ass) = association	1. Different population.<br>2. See different climates with contradictory outcomes.<br>3. Importance of climates in developmental phases and genders.<br>4. Validity measures in team setting.<br>5. Systematic review of intervention and qualitative work.<br>6. Actual experimental work.<br>7. Feedback and communication skills on these climates.<br>8. Logitudinal studies with goal orientations.<br>9. Behaviors assessment.<br>10. Individual but in a squad.
10	Jõesaar et al. (2011)	Estonia	Association between PMC, need satisfaction, intrinsic motivation and persistence.	424	Teens (11-16 Y)	Team sports	Competitive only at provicial level.<br>Engaged with their team (M-3.61 years)	Longitudinal	Peer MC,<br>Basic Psychological Needs,<br>Intrinsic motivation,<br>Persistence	AGT; SDT	1. Peer task MC indirectly affect intrinsic motivation and persistence via autonomy, competence, and relatedness needs.<br>2. Peer ego MC only negatively affect relatedness need.	1. Different age groups and target population, from different culture and competitive standards.<br>2. Control for covariance stability of psychological measure.<br>3. Other factors that lead to persistence and dropout.
11	JÕESAAR & Hein (2011)	Estonia	Association between parent, coach, peer MC, basic needs, and motivation	659 (F-230, M- 429)	Youth (9-17Y)	Team and individual	History 1-5Y	Longitudinal	Autonomy support from coach and parents, Peer MC, Basic Psychological Needs, Sport Motivation	SDT; AGT	1. Dropped out athletes had less need satisfactions, autonomy support from parents, intrinsic motivation, and peer task climate than persistent.	1. Longitudinal study with team and individual sports.<br>2. Multiple social environmental, and motivational variables among different competition levels.<br>3. Different age group and target population.
12	Jõesaar et al. (2012)	Estonia	Association between coach autonomy support, peer motivational climate and intrinsic motivation.	362 (F-110, M-252)	Teens (11-16 Y)	Individual sports and team sports	National and provincial level but not professional.	Longitudinal	Autonomy support from coach,<br>Intrinsic motivation	AGT; SDT	1. Autonomy support from coach and Task peer MC (+)= later intrinsic motivation over a training year.<br>2. Autonomy support from coach (Time 1) (+)= Task peer MC (Time 2), but not vice versa.	1. Parents influences on peer climate over time.
13	Ntoumanis et al. (2012)	UK	Association between coach, peer MC, moral attitudes, well-being and behavioral investment.	1: 267 (F-45)<br>2: 233 (F-53)<br>3. 149 (F-20)	Teens (12-16 Y)	Team sports	Competitive(training 3 hours per week)	Longitudinal	Moral Attitudes,<br>Emotional Well-/ill-being,<br>Intention/behavioral investment	AGT	1. Coach and Peer Task MC were more associated with adaptive outcomes than ego MC.<br>2. The predictive effects on outcomes varied across the time.	1. Find out why study variable varied over time.<br>2. Incorporating the parental MC.<br>3. Using objective markers of well-being, morality, and behavior in future longitudinal study.
14	Chan et al. (2012)	Hong Kong	Association between parent, coach, peer MC, effort, enjoyment, competence, and anxiety	408	Youth (9-18Y)	Individual sport	Youth sport	Cross-sectional	Parent, coach, peer MC,<br>Effort,<br>Competence,<br>Enjoyment,<br>Anxiety	AGT	1. Mother's impact was stronger for children than for adolescents.<br>2. Peers' impact was stronger for adolescents than for children.<br>3. Coach had an impact for both children and adolescents.	1. Measuring social influence.<br>2. Developmental change with potential moderators in a longitudnial design.
15	Atkins et al. (2013)	U.S.	Association between parents' and peers' motivational climate, self-esteem, sport compentece, and enjoyment	277 girls	Mean-12.7Y	Team and individual	Elite, select, and recreational	Cross-sectional	Parent support,<br>Peer task climate,<br>Competence,<br>Self-esteem,<br>Enjoyment,<br>Intention to continue	N/A	1. Parents Task MC (+)= enjoyment, self-esteem and competence.<br>2. Peer Task MC was not related to these outcomes.<br>3. Enjoyment (+)= intention to continue.	1. Older female or young male adolescents.<br>2. Longitudinal study.<br>3. Other psychological outcomes.<br>4. Measuring actual continuation.
16	Alcaraz et al. (2013)	Spain	Shortening questionnaires and validating	1: qual:17 M, quan: 114 (male 61%)<br>2. qual:15 (F-7, M-8)<br>quan: 309 (male 66%)<br>3. 204 (F-110, M-94)	1. qual (12-16Y<br>quan (10-19Y)<br>2. qual (10-12Y)<br>quan (11-19Y)<br>3. (9-18Y)	Team sports	Local and regional competition	Quantatative and qualitative; Measurement Development	Peer MCYSQ,<br>Sport climate,<br>Task and ego orientation	AGT; SDT	Development and Validation	1. Connect to multiple variables<br>2. Keep testing the quality of qustionnaires
17	Webb (2013)	Canada	To examine associations between peer climate and positive and negative affect	315 (F-129 M-186)	University student	Team sports	Recreational	Cross-sectional	Achievement goal orientation; positive and negative affect	AGT; SDT	1. Peer task climate +(=) positive affect.<br>2. peer ego climate +(=) negative affect.	1. Examine other positieve and negative outcomes.<br>2. Keep similar age but wider sample.<br>3. Professional sports.
18	Keegan et al. (2014)	N/A	Explore the constructions of motivational climates of three agents by athletes	28 (F-5, M-23)	Teens to Adults (15-28Y)	Individual and team sports	National and World class athletes	Qualitative	Constructions of motivational climates created by three social agents	AGT	1. The common themes from 2 and 3 social agents emerged (e.g., 3 agents: feedback/evaluative behavior).<br>2. Unique theme for each social agent also emerged (e.g., Parent: support and facilitation)<br>3. Complex contextual interactions between athletes behaviors and motivation.	1. Different methodology.<br>2. Gender, wider ethnicities and nationalities in larger qualitative way.<br>3. Provide environment that facilitates more in depth interview.
19	Hein & Jõesaar (2014)	Estonia	Association between Peer MC, autonomy support by coach and parents and Self-determined motivation	662 (F-221, M441)	Teens (11-16 Y)	Team sports	National and provincial level	Cross-sectional	Autonomy support from coach and parent, Sport motivation	AGT; SDT	1. AS from coach and parent (+)= self-determined motivation, but parent is stronger.<br>2. AS from coach and parent are associated with Peer MC, but coach was stronger.<br>3. Only intra-team competition of Peer ego MC (-)= self-determined motivation.<br>*AS: Autonomy Support	1. Additional connection between Self-regulation motivation and social contextual factors.<br>2. Drop out behavior.
20	Taylor et al. (2014)	UK(Wales and England)	Associations between physical self-concept, self-reported physical activity, and motivation, Peer MC and Teacher need support.	545 (51% F)	Children (10-12Y)	PE	PE	Longitudinal	Teacher Psychological Need Support,<br>Peer MC, Motivational Regulations, Physical self-concept, physical activity.	AGT; SDT	1. Physical self-concept and physical activity behavior decline during the transition period.<br>2. Teacher's need support +(ass) self-concept, Peer task climate +(ass) physical activity.<br>3. Teacher's influence was stronger than peer influence.<br>*(ass) = association	1. Objective measures of behavior.<br>2. Alternative aspects of school environment.<br>3. Conceptually distinguishing intrinsic and indentified regulations.
21	Atkins et al. (2015)	U.S	Associations between motivational climates created by 3 agents, goal orientation, sport competence, self-esteem, enjoyment and intention to continue	A :205 boys<br>B :200 boys	Children(8 Grade)	Individual and team sports	Youth sport	Cross-sectional	Parent, Peer and Coach MC, Goal Orientation, Sport competence, Self-Esteem, Enjoyment; intention to continue	AGT	1. 3 social agents task MC (+)= task goal orientation but parent and peer influence were more powerful than coach.<br>2. Task goal orientation (+)=competence, self-esteem, enjoyment.<br>3. Enjoyment, self-esteem (+)= intention to continue.	1. Explore older adolescents and racial/ethical minorities.<br>2. Longitudinal with actual participation.<br>3. Other psychological constructs.
22	Leo et al. (2015)	N/A	Association bewteen Motivational climate and antisocial behaviors	1,897 (F-519, M-1,378)	Teens (11-16 Y)	Team Sports	Youth Sport	Cross-sectional	Peer, Coach and Parent MC,<br>Antisocial behavior	N/A	1. Task MC by 3 agents (-) = Antisocial behavior.<br>2. Ego MC by 3 agents (+) = Antisocial behavior.<br>3. Parent Ego MC was the strongest predictor for antisocial behaivor.	1. Longitudinal study.<br>2. Using objective markers
23	Magbanua (2015)	U.S.	To examine associations between coach and peer climate, basic needs, and moral behaviors	185 (F-44, M-141)	14-18Y	Team and individual sports	Youth sport	Cross-sectional	Coacn and Peer MC; Basic psychological needs; moral behaviors and social desirability	AGT; SDT	1. Coach task +(=) autonomy, competence, peer task+(=) relatedness.<br>2. Coach, peer(also direct) -> prosocial behavior via autonomy need<br>3. Only coach ego -> antisocial behavior	1. Examine actual behavior with different variables<br>2. combine with longitudinal and qualitative
24	Greblo et al. (2016)	N/A	Associations between peer MC and perfectionism	274 (F-32%)	College students(18-25Y)	Individual and team sports	International, National, regional, college and recreational level	Cross-sectional	Perfectioninsm	AGT	1. Peer Task MC +(ass) Perfection striving<br>2. Peer Ego MC +(ass) Perfection concern<br>But, intra-team competition of peer ego MC +(ass) Perfection striving<br>*(ass)-> association	1. Younger and older participants.<br>2. Longitudinal + causal effects.<br>3. Parent and Coach MC.
25	Davies et al. (2016)	Canada	Asssociation between MC by 3 social agents and good and poor sport behaviors	244 males	Teens (11-17Y)	Team sports	Youth Sport(competitive)	Cross-sectional	Goal orientation,<br>Perceived ability,<br>Coach, parent and peer MC,<br>Good and Poor sport behavior	AGT	1. Coach and Parent influences were stronger than peers.<br>2. Dad task MC (+)= GSB for All ages.<br>3. Coach ego MC (+)= PSB for younger players.<br>4. Peer ego MC(+)= PSB for older players.<br>*PSB-Poor sport behavior, GSB-Good sport behavior	1. Pertinent items added to refind GSB and PSB.<br>2. Utilize four subscales or broaden scales.<br>3. Gender differences.<br>4. Improving communications with individual hockey associations.

26	Ettekal et al. (2016)	U.S.	Association between Peer MC and Empathic concern	665 (F-49.1%)	Teens (Mean-17)	Individual and team sports	Youth Sport(competitive)	Cross-sectional	Empathic concern	RDS(relational developmental system)	1. Peer Task MC (+)= empatic concern<br>2. Peer Ego MC was not associated with empathic concern.	1. Longitudinal.<br>2. More objective measure.<br>3. Include assessment points, multiple informants and various sports.
27	Webb & Forrester (2016)	Canada	Association between Peer MC and goal orientation	315	University students	Team sports	Intramural level	Cross-sectional	Goal orientation	AGT	1. Men -> higher Peer Ego MC than Women.<br>2. Task orientation +(ass) Peer task MC,<br>Ego orientation +(ass) Peer ego MC.<br>(ass)-> association.	1. Focus on youth ages of 12-17.
28	Duguay et al. (2016)	N/A	Develop, implement, and evaluate an athlete leadership program	27 female	Adults (18-27Y)	Team sports	Competitive level (varsity)	Intervention	Leadership behavior, Cohesion, Communication, Athlete satisfaction, Peer climate	N/A	1. The leadership program affected positively the human capitals (leadership behaviors).<br>2. The leadership program affected positively the social capitals (cohesion, communication, satisfaction, and peer motivational climate).	1. Large scale.<br>2. Male sample.<br>3. Control group.<br>4. Whether players intervene roles of others.<br>5. Teams not divided by tenure or mixed with another team.<br>6. Leadership program that teach specific behaviors to specific groups.
29	Tamminen et al. (2016)	Canada	Examining self- and interpersonal emotion regulation, and association with peer climate, sport enjoyment, and sport commitment	451 (F-204, M-247)	Youth (Mean-16.3)	Team sports	Competitive (M-7.82Y)	Cross-sectional	Intrinsic and Extrinsic Emotion Regulation;<br>Peer MC;<br>Sport enjoyment and commitment	ERS(emotion regulation strategy	1. Self-emotion regulation is highly associated with enjoyment and commitment.<br>2. In contrast, team-self regulation was less associated with enjoyment and commitment.<br>3. Peer task MC (+)= enjoyment and commitment, peer ego MC(+)= commitment.	1. Longitudinal and experimental study.<br>2. Larger sample to examine the interaction effect.<br>3. Different sport athletes.
30	Ingrell et al. (2016)	Sweden	To examine the relationships between ego peer climate, competence, and worry about sport performance.	64 (F-25, M-39)	Youth (12-13Y)	Team and individual sports	Student athlete	Longitudinal	Peer ego climate; Sport anxiety; Competence	AGT	1. Worry, peer ego climate increased while competence decreaesd over time.<br>2. competence -(ass) worry at the beginning.<br>Peer ego +(ass) worry.<br>*(ass)-> association	1. With a larger sample and more time points, logitudinal mediation and moderation between PMC and competence.<br>2. examine if anxiety is facilitating or debilitating.
31	Urquhart (2016)	U.S.	To examine motivational climate by coaches, peers, and team captains and associations with enjoyment, self-confidence, and resiliency.	95 (F-67, M-28)	13-22Y	Team and individual sports	Average (5years)	Cross-sectional	Coaches, peers, and team captains MC;<br>Self-confidence;<br>Enjoyment	N/A	1. Only captain task climate +(=) enjoyment<br>2. Coach task climate strongly related to enjoyment<br>3. Peer task climate +(ass) self-confidence<br>*(ass)-> association	1. Replicate it with bigger sample<br>2. Explore other factors with captain<br>3. Gender difference
32	Warburton (2017)	UK(England)	Association between Motivational climate by teachers and peers and achievement goal adoption	655 (F-296, M359)	Teens (11-14Y)	PE class	Recreational level	Longitudinal	Teacher and Peer MC,<br>2 by 2 goal adoption	AGT	1. Over the period,<br>Teacher Task MC increased while Peer Ego MC decreased.<br>2. There were mixed results in the relationship between MCs created by teacher and peer and goal orientations (e.g., teacher and peer task MC +(ass) performance avoidance goal).<br>*(ass)-> association	1. What are Congruent and incongruent Teacher and peer climate on motivation.<br>2. What role of teacher shape peer climate.
33	Schaillée et al. (2017)	Belgium	Association between Peer, Coach MC and positive youth development.	200 Females	Teens and adults (12-22 Y)	Team and individual sport(Urban dance and Martial Arts)	Recreational level	Cross-sectional	Coach and Peer MC,<br>Youth experience survey	AGT	PYD=positive youth development<br>1. Coach and Peer MC (+)= PYD<br>2. Coach task MC is very strong predictor of PYD<br>3. Peer task MC (+)= girls from non-migration family.	1. Objective markers.<br>2. Longitudianl work.<br>3. More appropriate procedure(based on social vulnerable situations).<br>4. Qualitative approach
34	Çağlar et al. (2017)	Turkey	Associations between Coach, Peer, and Parent MC and dispositional flow	220 (F-86, M-134)	Mean-14.24	Team Sports	Competitive (National League)	Cross-sectional	Coach, Peer, Parent MC; dispositional flow	AGT	1. Coach, peer, parent task MC (+) = dispositional flow	1. Experimental and Longitudinal for causal relationship.<br>2. Qualitative approach and compare age.
35	Mclaren et al. (2017)	Canada	Association between Peer MC and group cohesion	355<br>(F-14 teams, M-13 teams)	Youth (10-17 Y)	Team sports	Competitive level	Longitudinal	Group cohesion	AGT	1. Peer climate was highly related to task cohesion.<br>2. Peer task climate maintained over time.<br>3. Any of Peer MC ≠ social cohesion.	1. Experimental design for surrounding nature of influence between the two constructs.<br>2. Increasing collection period, better insight into temporal influence.<br>3. Recreational sport or adult sports.
36	Beck et al. (2017)	U.S.	Association between Parent, coach, peer MC, goal orientation and mental toughness	599 (M-309, F-290)	Mean-15.90(F)<br>16.46(M)	Team and individual	High school varsity	Cross-sectional	Parent, coach, peer MC<br>Mental toughness	AGT	1. Parent, coach, and peer Task MC (+)= Task goal orientation.<br>2. Parent, coach Ego MC (+)= Ego goal orientation.<br>3. Task goal orientation (+)= mental toughness.	1. Longitudinal Study.<br>2. Extend to elite athletes.
37	Agans et al. (2018)	U.S.	Assciation between Peer MC and character profiles	655 (45% female)	High school teens (Mean-16)	Individual and team sports	Youth sport	Cross-sectional	Peer MC, Improving the self, Improving teammates, Improving the game	N/A	Athletes in peer task climate were most likely to demonstrate the positive character attributes.	1. Longitudinal.<br>2. Other features of team environment.<br>3. Objective measure (observation).
38	Chu (2018)	U.S.	Explore the motivational processes from coaches, peers, and parents, and association with need satisfaction, motivation, vitality, burnout, and drop out.	Quantitative: 322<br>Qualitative: 37	9th-12th grades	Team and individual	Varsity and non-varsity	Mixed methods (quantitative+ qualitative)	Coach, peer, and parent MC;<br>Need satisfaction, frustration,<br>Motivational regulations, subjective vitality, burnout, intention to drop out	SDT; AGT	1. Positive social environments and need satisfaction=(+) autonomous motivation and well-being<br>2. Negative social environement and need frustraction=(+)controlled and amotivation and ill-being<br>3. Among social agents, coach (strongest) was protective and risk to burnout and dropout.	1. Longitudinal Study.<br>2. More evenly distributed sample.<br>3. Different climates.<br>4. Forming homogenous focus groups who are not friends with each other.
39	Gómez-López et al. (2019)	Spain	Association between Peer, coach MC and fear of failure	479 (F-229, M-250)	Teens (16-17Y)	Team sports	Competitive level	Cross-sectional	Peer MC, Coach MC,<br>Fear of failure	AGT	1. Both Coach and Peer Ego climate (+)= fear of failure.<br>2. Mostly, less experienced players felt task-climate, experienced players felt ego-climate.	1. Other levels of sport performance in other categories.<br>2. Parents' influence on fear of failure.
40	Mellano (2019)	U.S.	1. Association between Peer MC and Well-being based on peer relationship profiles.<br>2. Relationships between Coach and Peer MC and engagment.	1: 245 (F- 49.2%, M-50.2%)<br>2: 255 (Female)	Teens (14-18Y)	Team sports	Competitive Youth	Cross-sectional	1: Peer MC, Peer Acceptance, Friendship Quality and Conflict, Sport Enjoyment, Sport Anxiety, Athlete Burnout<br>2: Coach, Peer MC, Engagement, Effor, intention to continue	AGT	1. Based on 5 peer relationship profiles, different peer MC emerged and relationships with well-being also differed.<br>2. Peer and Coach task MC were +(ass) adaptive outcomes,<br>Peer and Coach ego MC do not always (ass) negative outcomes.<br>*(ass)-> association	1. Assessing multiple times.<br>2. Find what makes item unreliable.<br>3. Different sports, gender, age, and race.<br>4. Diverse outcome variables.
41	Cheon et al. (2019)	South Korea	Association between autonomy supportive teaching, peer climate, and prosocial behaivor through intervention	42 PE Teacher(F-10, M-32),<br>2219 students(F-1429, M-1310)	Teacher (28-45Y),<br>Students (13-18Y)	PE	PE	Intervention	Autonomy support and controlling teaching;<br>Need satisfacation and frustration;<br>Peer MC;<br>Pro and antisocial behavior	SDT; AGT	AST: Autonomy supportive teaching<br>1. AST enhanced perceived AST, need satisfaction, peer task MC, prosocial behavior, and academic success.<br>2. AST diminished percevied controlling teaching, need frustration, peer ego MC, and antisocial behavior.	1. Adding objective ratings<br>2. Having teachers in control group complete an active 3-part intervention (unrelated to motivating style).<br>3. Including baseline scores of rater-observed teaching styles.
42	Ingrell et al. (2019)	Sweden	To examine achievement goals and perceived motivational climates by coaches, peers, and parents	78 (F-30, M-30)	Youth (Mean-12)	N/A	Student athlete	Longitudinal	Coach, peer and parents MC; task and ego orientations	AGT	1. Both task and ego orientation decresased over time.<br>2. Peer task +(=) task orientation<br>coach ego +(=) ego orientation	1. Distiontion between training and competition.<br>2. Larger sample size.<br>3. Examining similarities and differents between cohorts.
43	Dandan (2019)	U.S.	To examine non-targeted effects of Connect Through PLAY (intervention)	138 (F-55%)	10-17 Y	physical activity (after school)	Physical activity (after school)	Intervention	Internalizing features; physical activity; self reported climate; observed climate	AGT; SDT	1. Intervention groups showed decrease in internalizing symtoms in post.<br>2. social factors (including peer cliamate)≠ internalizing symtomes.<br>3. change in internalizing symtoms +(=) change in inclusive climate.	1. Different types and sources of social support.<br>2. Gender.<br>3. Other youth characteristics.<br>4. Larger sample.<br>5. Additive impact (e.g., parent support).
44	Girard (2020)	N/A	Associations between contesting orientation, peer motivational climate, and moral disengagement	237 (F-57, M-180)	Youth (high school, Junior varsity and varsity)	Team sports	Junior varsity and varsity.	Cross-sectional	Moral disengagement, contesting orientation, peer MC,	RDS(relational developmental system)	1. There are mixed results between contesting orientations and moral disengagement.<br>2. Peer Task MC (+)= moral disengagement.<br>3. Contesting orientation and moral disegagement did not differ by Peer MC.	1. Different sports<br>2. Longitudinal.<br>3. Causality and different time points.<br>4. Measuring at the team level.
45	Rodrigues et al. (2020)	Portugal	To examine the associations of learning and performance MC by teachers and peers on PE grades with need satisfaction as a mediator.	589 studenst (F-390)	10-18 Y	PE	PE	Cross-sectional	Learning and Performance Orientation in Physical Education Classes;<br>Basic Needs Exercise Scale; PE grades	AGT; SDT	1. Learning MC by teachers, peers (+)-> three needs but competence (stronger). Also performance teacher MC (-)-> three needs.<br>2. Teacher, peer Learning MC indirect (+)-> PE grades.<br>3. Peer performance (+)-> competence and autonomy needs , indirect (+)-> PE grades.	1. Future studies should test between groups.<br>2. Using qualitative or mixed-method, longitudinal or intervention designs<br>3. Mediation analysis using other motivational constructs.<br>4. Using 2*2 model for further examination.
46	Mossman et al. (2021)	UK(England)	Associations between Motivational climate by three agents and life skill development	308 (F-63, M-245)	Teens and adults (11-21 Y)	Team sports	Not competitive	Cross-sectional	Peer, Coach, Parent MCs, Life Skills	AGT	1. Peer(strongest), coach, and parent Task MC were +(ass) life skills.<br>2. Coach, and parent(strongest) Ego MC were (-)= life skills.<br>3. Peer ego MC (+)= a few life skills.	1. Perspective of coach and parents for sporting climate and life skill development.<br>2. Experimental and longitudinal study.<br>3. Cluster analysis.<br>4. MC by parents based on age of participants and influence outside of sports.<br>5. Separating father and mother's influence.<br>6. A huge data analysis for potential variable.<br>7. Include male and female.
47	Isoard-Gautheur et al. (2021)	N/A	Associations bewteen Peer MC, motivation and sport-related well-being.	73 (F-31, M-42)	Adults (18-25Y)	Team and individual sports	Competitive	Longitudinal	Peer MC,<br>Motivation,<br>Engagement,<br>Burnout	AGT; SDT	1. Peer Task MC (+)= autonomous motivation (+)= engagement.<br>2. Peer Ego MC (+)= controlled motivation (+)= burnout.<br>Autonomous and cotrolled motivations were mediators.	1. Discrepancy between climate by three agents.<br>2. Potential mediations.<br>3. More direct method<br>(e.g. neurophysiological measures).<br>4. Longer period
48	Weiss et al. (2021)	U.S.	Association between coach, peer MC, and group cohesion	235 female	Teens (14-18Y)	Team sports	Competitive level<br>(junior olympic)	Cross-sectional	Coach MC,<br>Peer MC,<br>Group cohesion	AGT	1. Coach and peer Task MC +(ass) team cohesion.<br>2. Peer ego(conflict) MC -(ass) team cohesion.<br>*(ass)-> association	1. Longitudinal and mixed methods.<br>2. Gender difference.<br>3. Moderators (win/loss, perceived success, starter/non-starter)
49	Kavussanu & Ring (2021)	N/A	Examining morality in Peer MCs, and in moral disengagement and empathy.	Study1(club): 190<br>Study2(school): 296 (F-306, M-296)	Youth (12-18Y)	Team(football)	History<br>Study1: M-6.78Y<br>Study2: M-5.3Y	Cross-sectional	Pro and anti social behavior,Peer MC,<br>Moral disengagement,<br>Empathy	N/A	1. Moral behaviors varied across the contexts and ages.<br>2. Peer task MC =(+) prosocial behaviors.<br>2. Moral disengagement was related to antisocial behaviors.	1. Exmaining other sport teams and types.<br>2. See different degree of physical contact and difference between team and individual.<br>3. Longitudinal design.<br>4. Determining how athletes who identify highly with their team behave to team and opponents.
50	Rillo-Albert et al. (2021)	Spain	To examine the effect of the pedagogical model and on relational well-being.	287	3th, 4th secondary school	PE	PE	Intervention	Teacher intervention;<br>Peer MC	AGT; SDT	1. Relational well-being increased in the post.<br>->Peer task climate increased and peer ego climate decreased in the post.	1. More number of schools with longer measurement.
51	Tidmarsh et al. (2022)	UK	Exploring Peer Motivational climate	S1: 30 Female<br>S2: 43 Males	Teens<br>(7-11 school year)	PE class and team sports	Recreational level	Qualitative	Peer motivational climate	AGT	1. Girls mostly perceived teacher and peer Ego MC but feel enjoyment for PE classes.<br>2. Boys suggest classes set by ability rather than gender to support each other.	1. Students' own motivational climate.<br>2. How the future intervention is best devliered.
52	McCann et al. (2022)	U.K.	To examine the perceived motivationally-relevant behaviors and influences of social agents for football players	8 athletes,<br>4 parents	athletes (M-18.5)	Team sports (football)	Competitive (13 y of trainings)	Qualitative (retrospective)	Motivational relevent perceptions	AGT; SDT	Coach and Peers are becoming more important as their athletic devleopment progresses while parents are not as important as before.	1. Different sports and contexts.<br>2. Different social agents with interventions.<br>3. Identify unique motivational influence by social agents.<br>4. Longitudinal.

53	Habeeb et al. (2023)	U.S.	Asscoiation between parent, coach, peer MC and burnout and engagement	130 (F-65, M- 85)	Teens (14-19Y)	Individual and team sports	Competitive level	Cross-sectional	Coach, Parent, Peer MC, Burnout, Engagement	AGT	1. Parents, coaches, and peers had consistent influences on burnout and engagement.<br>2. Parents influnce were less than coaches and peers.	1. Longitudinal design.<br>2. Ideographic approaches.<br>3. Team level.
54	McLaren et al. (2023)	Canada	Associations between Peer MC, mental health and adherence	130 (F-44, M-86)	Youth (Mean-13)	Team sports	Competitive level	Cross-sectional	Peer MC<br>Well-being<br>Intention to return to the team	AGT	1. Peer task MC (+) = Well-being and intention to return<br>2. No relationship found with Peer Ego MC.	1. Longitudinal study.<br>2. Extending to other competitive and recreational sports.<br>3. Interactive effects of climates and find "optimal mix" of climates.
